# Internet of Things Node with Real-Time LoRa GEO Satellite Connectivity for Agrifood Chain Tracking in Remote Areas

**DOI:** 10.3390/s25206469

**Published:** 2025-10-19

**Authors:** Giacomo Giannetti, Marco Badii, Giovanni Lasagni, Stefano Maddio, Giovanni Collodi, Monica Righini, Alessandro Cidronali

**Affiliations:** Department of Information Engineering, University of Florence, Via di Santa Marta 3, IT-50139 Florence, Italy; marco.badii@unifi.it (M.B.); giovanni.lasagni@unifi.it (G.L.); stefano.maddio@unifi.it (S.M.); giovanni.collodi@unifi.it (G.C.); monica.righini@unifi.it (M.R.); alessandro.cidronali@unifi.it (A.C.)

**Keywords:** agrifood chain traceability, direct-to-satellite IoT, GEO satellite, Internet of Remote Things, Internet of Things, long-range frequency hopping spread spectrum, LoRa, satellite communications, wireless sensor networks

## Abstract

This work presents an Internet of Things (IoT) node designed for low-power agrifood chain tracking in remote areas, where long-range terrestrial communication is either unavailable or severely limited. The novelty of this study lies in the development and characterization of an IoT node prototype that leverages direct-to-satellite connectivity through a geostationary Earth orbit (GEO) satellite, using long-range frequency-hopping spread spectrum (LR-FHSS) modulation in the licensed S-band. The prototype integrates a microcontroller unit that manages both the radio modem and a suite of sensors, enclosed in a plastic box suitable for field deployment. Characterization in an anechoic chamber demonstrated a maximum effective isotropic radiated power (EIRP) of 27.5 dBm, sufficient to establish a reliable satellite link. The onboard sensors provide global positioning as well as measurements of acceleration, temperature, humidity, and solar radiation intensity. Prototype performance was assessed in two representative scenarios: stationary and mobile. Regarding energy consumption, the average charge drained by the radio modem per transmission cycle was measured to be 356 mC. With a battery pack composed of four 2500 mAh NiMH cells, the estimated upper bound on the number of transmitted packets is approximately 25,000.

## 1. Introduction

### 1.1. Background and Contributions

Precision agriculture (PA), also referred to as smart farming, aims to improve crop yield and process efficiency while enhancing sustainability [1]. Despite its name, PA is not only limited to soil cultivation, but also encompasses livestock breeding and farming. In recent years, particular attention has been devoted to the traceability of agrifood chain processes [2]. Institutional interest in PA is evidenced by ongoing COST Actions (https://www.cost.eu/ (accessed on 4 August 2025)) promoted by the European Union, as well as by the Next Generation EU (NGEU) program, which supports the Italian National Recovery and Resilience Plan (NRRP). The NRRP, in turn, includes the creation of National Research Centers, such as the one dedicated to Agricultural Technologies (AgriTech) [3]. In this context, the Internet of Things (IoT) paradigm is pivotal for gathering substantial data from numerous distributed sensors and devices [4] from a long range (greater than tens of kilometers) [5]. This setting constitutes a so-called wireless sensor network. While sensor sets and devices are tailored to specific applications, IoT node architectures are typically built around a low-power microcontroller and a radio modem for data exchange with cloud storage. For data communications, IoT nodes generally employ either low-power, low-data-rate terrestrial links or direct-to-satellite (DtS) technologies, the latter being particularly advantageous in remote areas (Internet of Remote Things). Application scenarios where satellite connectivity is especially beneficial are discussed in [6,7,8].

Satellite-assisted IoT applications are often designed to exploit low Earth orbit (LEO) constellations, primarily because of the lower propagation delay and reduced link budget requirements compared to geostationary Earth orbit (GEO) systems. GEO satellites, however, offer continuous visibility, as they remain fixed relative to the Earth. By contrast, although LEO satellites are closer to the Earth’s surface, their orbital motion requires Doppler-shift correction, frequent handovers, and—with sparse constellations—significant delays may occur before the next satellite becomes available. Despite their higher link budget demands, GEO satellites are particularly advantageous for time-critical applications, since they ensure uninterrupted connectivity. For both LEO and GEO constellations, long range (LoRa) technology (https://www.semtech.com/lora (accessed on 4 August 2025)) enables reliable IoT communications, even in remote areas where other radio access technologies are unavailable or ineffective. This capability is especially valuable in scenarios such as free-range cattle farming in mountainous regions, where animals often graze beyond the coverage of terrestrial networks.

In this context, the novelty and research objectives of the proposed work are as follows:

- The development and characterization of an IoT sensor node based on LoRa connectivity supported by GEO satellite radio access technology. The node integrates relevant sensors and a LoRa satellite modem. Although the design was tailored for cattle breeding [9], the architecture can be adapted to other use cases.
- This work extends the proof-of-concept from [10] by presenting a more mature prototype enclosed in a dedicated box suitable for field testing in realistic scenarios. The experimental characterization addresses the entire end-to-end process, from data acquisition on the field to data availability in cloud storage.
- The evaluation of real-world experimental tests with GEO connectivity, enabling a performance assessment of the proposed IoT node in terms of power consumption.

### 1.2. Literature Review

PA relies on several enabling technologies, including remote sensing, the IoT paradigm, autonomous systems, prediction and decision-making techniques, and global navigation satellite systems (GNSSs). An overview of sensors and remote sensing technologies for agriculture is provided in [11], which enumerates a variety of techniques such as thermal imaging, multispectral imaging, radar, light detection and ranging (LiDAR), and aerial or unmanned aerial vehicle (UAV) imaging. Within this framework, the role of IoT in agriculture is discussed in [12], which reviews IoT sensor technologies applied to PA. An IoT-based approach for plant growth control is presented in [13], while a similar review dedicated to livestock applications is reported in [14]. Furthermore, Ref. [12] provides a comprehensive analysis of key IoT parameters relevant to PA.

Regarding IoT connectivity, recent studies have investigated the use of satellite links [15], with particular emphasis on LoRa technologies for LEO satellite IoT nodes [16]. However, field-tested use cases of DtS IoT nodes remain limited. For instance, Ref. [17] reports the experimental characterization of an IoT device based on LEO satellite communications for monitoring athletes in extreme environments. The system relies on a constellation of more than 170 CubeSats, which nonetheless does not ensure continuous coverage. To enable DtS satellite connectivity and increase the network capacity, Semtech has introduced the long-range-frequency hopping spread spectrum (LR-FHSS) [18,19]. This modulation permits low data rate IoT applications with thousands of uplink devices (from Earth to satellites) [15,20]. Such features are unfamiliar to traditional satellite networks, as they are primarily designed for high-throughput data applications in broadcast downlink. To the best of the authors’ knowledge, this is the first scientific work reporting on a GEO satellite-enabled IoT node, validated through on-the-field experimental tests.

The paper is organized as follows. The IoT node architecture is described in Section 2. There, the components of the IoT node are described, the cloud service and Python interface are outlined, and the assembled IoT node is shown. The results in terms of effective isotropic radiated power (EIRP), on-the-field tests, and related energy consumption are presented in Section 3 and discussed in Section 4. Eventually, the conclusions are drawn in Section 5.

## 2. Materials and Methods

**Figure 1:**
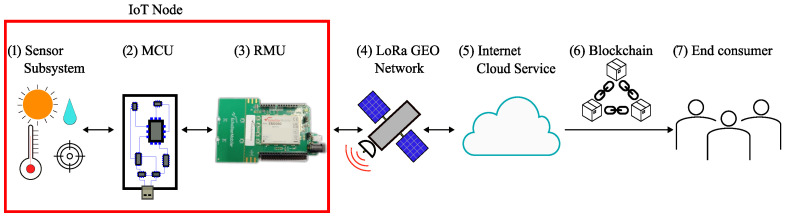
Conceptual block diagram of the proposed LoRa GEO agrifood chain tracking architecture. The components of the IoT node are included within the red rectangle.

The conceptual block diagram of the proposed LoRa GEO agrifood chain tracking architecture is depicted in Figure 1.

The IoT node—within the red rectangle in Figure 1—is composed of the sensor subsystem (1), the microcontroller unit (MCU) (2), and the radio-modem unit (RMU) (3). With reference to Figure 1, the MCU (2) acquires data from the environment in which the IoT node is located, thanks to the sensor subsystem (1), and drives the RMU (3). This eventually becomes the hardware in charge of transmitting the data to the GEO satellite (4); data transmission then takes place via real-time—that is, the system guarantees responses within specified deadlines [21]—satellite LoRa communication. The GEO satellite receives the data packets and sends them back to Earth, where they are made available via an internet cloud service (5). Data are then processed, for instance via blockchain (6) for traceability and integrity [22,23], and made available to the end consumer (7). Steps (6) and (7) are outside of the scope of this work.

This section proceeds with the following structure: Section 2.1 details the IoT node’s hardware components, system integration, and enclosure, Section 2.2 covers the satellite budget uplink, and Section 2.3 outlines the cloud service interface terminal.

### 2.1. IoT Node

In this subsection, the hardware components are first described, followed by their integration within the prototype, and finally the housing enclosing the hardware.

#### 2.1.1. Hardware Components

The IoT node’s hardware, which can all be purchased commercially, consists of the following:

- Sensor subsystem
  – GNSS module
  – accelerometer
  – temperature and humidity sensor
  – solar radiation intensity sensor
  – *current probe* (for monitoring purposes only)
- MCU
- LoRa RMU

The list above includes the sensors that make up the sensor subsystem [(1) in Figure 1]. The sensors actually used depend on the specific use case; e.g., temperature is common to every application, humidity sensors may be used for soil moisture in crop monitoring, while accelerometers and GNSS are commonly used for cattle breeding. In particular, the use of GNSS for cattle breeding is established for tracking [24], pasture management [25], grazing habits [26], and the environmental impact of grazing [27]; while an accelerometer is commonly adopted to monitor cattle position and activity, such as ingestion, lying down, standing, walking, and estrus [28]. Ambient temperature and humidity are useful, for example, in assessing cattle-induced estrus [29]. The solar radiation intensity was also measured, as it affects cattle behavior [30].

Regarding the current probe, power consumption plays an important role in IoT devices [31,32]. The monitoring of the RMU supply current was implemented in the proposed IoT node to estimate the power consumption and, consequently, the battery life. Although sensors and the MCU can be optimized for low-power operation, the RMU’s power consumption is proportional to the EIRP required to establish a successful communication. For this reason, the RMU is the most power-hungry component of the IoT node, and therefore, only its current consumption was monitored. The EIRP requirements for GEO satellite networks are described in Section 2.2. The current probe is used only during the experimental characterization of the IoT node, but is not expected to be deployed in the final implementation.

##### GNSS Module

In this work, we adopted a GNSS module from Grove, a trademark of Seeed Technology Co., Ltd., located in Shenzhen, China, employing the Air530 module, working with GPS, Beidou, Glonass, Galileo, and QZSS constellations. The module communicates with the MCU using a UART interface and is specified for typical and maximum working currents of 30 mA and 60 mA, respectively. To save power, the GNSS module can be set in sleep mode. The coordinates provided by the GNSS module are in the format: degrees, primes, decimal of primes. The GNSS module acquires the position every second with a precision of 1.1 m on the Earth’s surface. The module is connected to a ceramic antenna via a micro-sized coaxial cable. The dimensions of the GNSS module and the antenna are 42 mm × 24 mm and 15 mm × 15 mm, respectively.

##### Accelerometer

The IoT node integrates a three-axis low-power accelerometer, based on the LIS3DH integrated circuit (IC) from ST Microelectronics, located in Plan-les-Ouates, Geneva, Switzerland. The accelerometer communicates with the MCU through an I2C serial interface. The current consumption is 150 μA in measurement mode and to 21 μA in standby mode. The module dimensions are 23.8 mm × 20 mm.

##### Temperature and Humidity Sensor

The temperature and humidity sensor is based on a DHT22 module, which is a basic, low-cost digital temperature and humidity sensor. It integrates a capacitive humidity sensor and a thermistor. The data are made available through a single-wire serial interface to the MCU. According to the datasheet, the accuracy is ±0.5 °C for temperature and ±5% for relative humidity measurements. The maximum current consumption is 1.5 mA during measurements and 50 μA in standby mode, making this sensor an attractive choice for battery-operated systems. The sensor dimensions are 41.5 mm × 22 mm.

##### Solar Radiation Intensity Sensor

**Figure 2:**
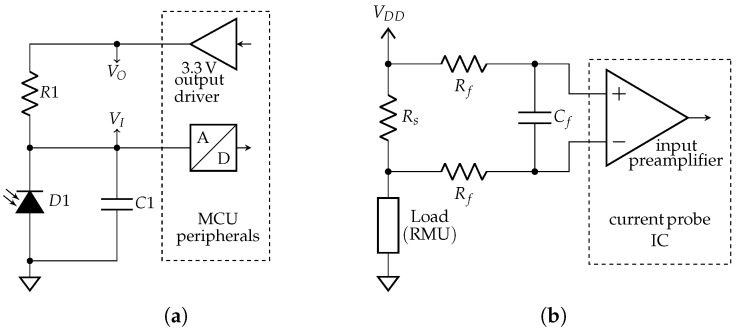
Circuit schematics: (**a**) solar radiation intensity sensor (R1=5.6 kΩ and C1=10 nF); (**b**) current probe ($R_{s}=100\;m\Omega$, $R_{f}=100\;\Omega$, $C_{f}=14\;mF$, and $V_{DD}=3.3\;V$).

The Amphenol SUF083J001 integrates a reverse-biased photodiode cell to measure the intensity of solar radiation, providing an output current linearly proportional to the luminous flux. The manufacturer is Amphenol and it is located in Wallingford, Connecticut, USA. The conversion factor from current to intensity of solar radiation is 5.2 × 10^4^ lx/mA. The biasing circuit is shown in Figure 2a. The output current is 0.5 mA at a solar radiation of 2.6 × 10^4^ lx of a 2856 K CIE standard illuminant A. Considering a biasing resistor R1=5.6 kΩ, the voltage at the input of the ADC ranges between 0.5 V and 3.3 V. The module dimensions are 20 mm × 20 mm × 17 mm. The relative sensitivity relationship versus wavelength and the radiation pattern are provided in the datasheet.

##### Current Probe

The probe currently uses a Gravity I2C digital wattmeter module based on the Texas Instruments INA219 IC. The manufacturer of the IC is Texas Instruments and is located in Dallas, Texas, USA; on the other hand, the manufacturer of the wattmeter is DFRobot, a company headquartered in Shanghai, China. It connects in series with the RMU power supply and determines current by the voltage drop over a 100 mΩ shunt resistor. An RC filter is included to reduce digital noise, as shown in Figure 2b.

##### Microcontroller Unit

The MCU is a ST Nucleo 144 evaluation board, based on an ST Microelectronics STM32 ARM microcontroller. The MCU is manufactured by ST Microelectronics, located in Plan-les-Ouates, Geneva, Switzerland. The requirements for the MCU unit depend on the complexity of the sensor subsystem and the processing requirements. In the authors’ opinion, the proposed selection is adequate for most agrifood traceability applications. The MCU has a maximum working current of 500 mA, mainly depending on the number of employed output lines, with a supply voltage range between 4.75 V to 5.25 V. The printed circuit board (PCB) dimensions are 133.3 mm × 70 mm. Supplementary Material about the MCU firmware of the IoT node is available in [33].

##### LoRa Radio-Modem Unit

The EchoStar EM2050 (https://echostarmobile.com/product/em2050-evk-evaluation-kit/ (accessed on 4 August 2025)) radio-modem constitutes a commercially available technological solution for IoT connectivity with GEO satellites. The RMU manufacturer is EchoStar Mobile Ltd and is located in Milton Keynes, United Kingdom. Despite their longer propagation delay with respect to low- and medium-Earth orbit satellites, the GEO satellites enable real-time connectivity between the sensors and the cloud-based service, as the IoT node and the gateway are always within the coverage area of the satellite. The RMU provides all functions required for the GEO satellite connection. It is equipped with two surface-mount ceramic S-band antennas (M310220 chip antenna from Kyocera/AVX, manufactured by Kyocera Corporation, located in Kyoto, Japan, Tx 1980 MHz to 2020 MHz, Rx 2170 MHz to 2200 MHz, linear polarization, maximum input power of 0.5 W=27 dBm). The RMU communicates through a UART serial interface to the MCU. The current consumption for the RMU depends on the mode, state, and operation performed. Table 1 reports the measured current for each RMU state. The joining power corresponds to an antenna input power of 27 dBm, that is, the maximum input power rating for the adopted antennas. The RMU can be supplied with 3.3 V or 5 V; the former is preferred, since it avoids the internal switching voltage regulator, thus reducing power loss. The dimensions of the PCB are 107.5 mm × 69 mm.

The RMU is based on the LR1120 transceiver from Semtech Corp (https://www.semtech.com/products/wireless-rf/lora-edge/lr1120 (accessed on 4 August 2025)). Such a transceiver provides multi-band communication with LoRa and LR-FHSS radio access technology over sub-GHz, 2.4 GHz industrial, scientific and medical (ISM) bands, as well as licensed S-Band for satellites, as the one employed in the present work.

The LR-FHSS modulation is for low data rate and is described in detail in [18,19]; the reader can find a comparison of different upload transmission approaches for the DtS use case considering basic LoRa and LR-FHSS in [34]. The parameters for LR-FHSS here used are reported in Table 2. The RMU changes adaptively between DR8 and DR9.

#### 2.1.2. System Integration

Table 3 reports the maximum supply current of each hardware component in the IoT node. The MCU supply current is largely driven by the debug circuitry, which can be removed in a final commercial design, thereby significantly reducing the MCU’s energy demand. The RMU in its network-joining state exhibits the second-highest current consumption, as it operates at maximum transmission power in this state.

**Figure 3:**
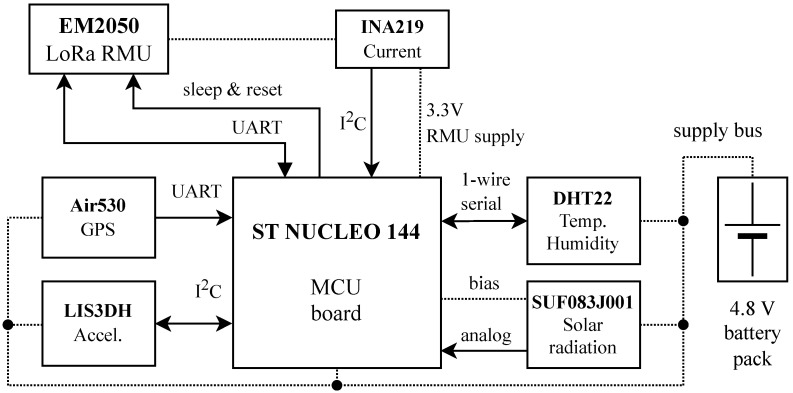
Hardware block diagram of the IoT node, highlighted in the red box of Figure 1. Solid lines indicate data lanes and flow direction, dotted lines indicate DC power distribution.

The detailed block diagram of the proposed IoT node is illustrated in Figure 3. The interfaces between the MCU and the peripherals are shown together with the power supply. The latter is provided by a battery pack composed of four 2500 mAh NiMH rechargeable batteries. The battery pack provides a nominal voltage of 4.8 V and is connected to the MCU board, which in turn distributes the supplies to the other peripherals.

**Figure 4:**
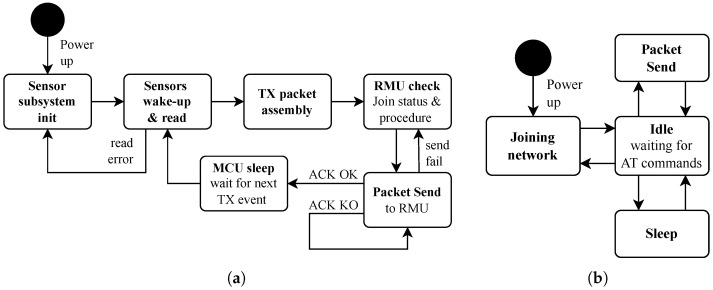
State diagrams: (**a**) MCU firmware and (**b**) RMU operations.

At power-up, the MCU initializes its core peripherals and the external sensor subsystem; then the main cycle starts, where the sensors are periodically interrogated and the acquired data are transmitted to the satellite gateway. Figure 4a depicts the firmware state diagram of the MCU.

First, all the employed sensors in the subsystem are woken up and read sequentially; the sensor data are then encoded, and the TX payload is assembled. At this time, the *packet send* command is issued to the RMU; the MCU checks for the acknowledgment ACK response for successful communication and repeats the *packet send* command if the ACK check has failed. In case of repeated failures, the RMU is interrogated for network join status by the MCU, and the join procedure is reissued if necessary. After a successful data transmission, the MCU waits for the next acquisition cycle; the firmware was designed to drive the peripherals and sensor subsystem in low-power sleep mode when the latter are not operative. The sensors can also be turned off in hard mode by removing the supply bias.

The sleep operation is particularly important for saving battery power, especially considering the RMU. The latter is set in sleep state when the node is not transmitting. Even during sleep, the real-time GEO satellite connection is preserved, eliminating the need for a new joining upon waking up, which represents a significant advantage compared to an LEO satellite connection.

**Figure 5:**
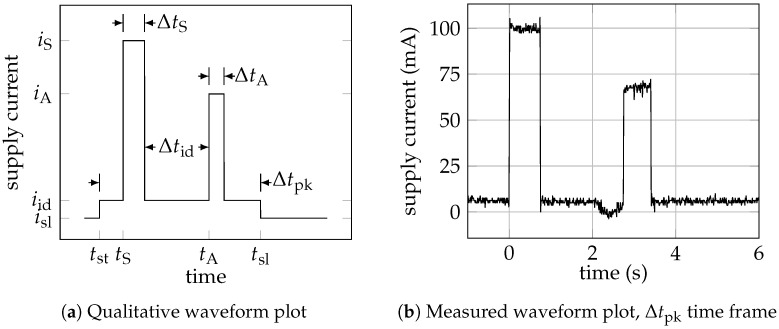
RMU supply current during a single packet transmission: (**a**) qualitative waveform (see Table 1 for current values); (**b**) instance of a measurement trial at $P_{TX}=15\;\mathrm{dBm}$, data from [10].

The supply current waveform for the RMU is shown in Figure 5, where a message send operation is performed. The cycle starts at $t_{\mathrm{st}}$ with the RMU in sleep mode (current $i_{\mathrm{sl}}$). Then, the RMU is woken up in the idle state (current $i_{\mathrm{id}}$) before transmitting the data, the latter starting at $t_{S}$ instant (current $i_{S}$) and lasting $\Delta t_{S}$. Quantity $\Delta t_{S}$ is called time on air. After data transmission, the RMU returns to the idle state, for $\Delta t_{\mathrm{id}}$, awaiting the ACK message. The latter operation lasts for $\Delta t_{A}$, and requires a second burst of current ($i_{\mathrm{ACK}}$). After that, the RMU returns to the idle state and then to the sleep state by the MCU command, eventually ending the cycle at $t_{\mathrm{sl}}$. When the IoT node is turned on at start-up, the RMU joins the satellite network and then enters the above cycle indefinitely. In Figure 4b, it is possible to see such a state diagram. In this procedure, energy consumption is maximum at initialization. In [32,35], details on the RMU current profile are given.

#### 2.1.3. Prototype Enclosure

**Figure 6:**
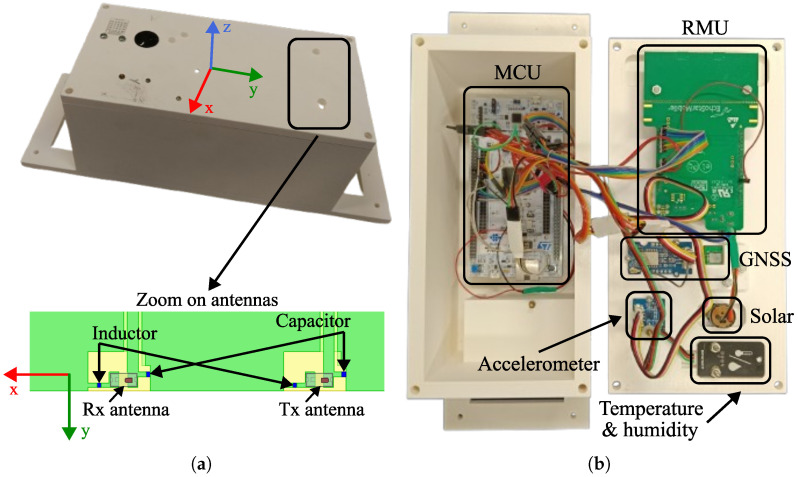
IoT node: (**a**) closed realized prototype with zoom on the RMU antennas from the CAD model; (**b**) open realized prototype. Enclosure box sizes are 108 mm × 110 mm × 320 mm.

For a mechanically robust IoT node prototype, a plastic enclosure was designed to accommodate all electronic components. The design paradigm places the MCU and the battery pack at the bottom of the box, while the remaining peripherals are mounted on the lid [Figure 6]. Spacers are used to secure the MCU and the RMU, whereas the battery pack is housed in a dedicated compartment with its own cover. At this stage of prototype development, all components are interconnected using wires rather than integrated on a PCB. The current focus is to demonstrate the design and characterization of an IoT node equipped with satellite connectivity; the integration of the MCU and peripherals on a single PCB is reserved for a future development stage. In this preliminary design, no specific measures were taken to protect the hardware from environmental or mechanical stresses.

Regarding the ceramic antennas for the RMU [Figure 6], the documentation recommends mounting them on the top side of the enclosure, keeping them at least 3 mm away from the walls to prevent detuning; avoiding external objects that obstruct the line of sight between the IoT node and the satellite is also recommended. To avoid antenna detuning, spacers are used to support the RMU. In addition, a slot was created in the lid to increase the distance from the antenna. Regarding the ceramic antenna of the GNSS module, it was installed on the back of the box cover.

The solar radiation sensor was installed in a recess of the box cover, such that it could be exposed directly to the environment. In the same way, a window on the box cover was opened to host the temperature and humidity sensor [Figure 6]. The designed box has slots to allow it, in a next phase of the project, to be attached to the collars of the animals. The dimensions of the box are 108 mm × 110 mm × 320 mm with slots and 108 mm × 110 mm × 220 mm without slots. The box was 3D-printed using acrylonitrile butadiene styrene (ABS) filaments and fused deposition modeling as a printing technique. The CAD files of the box are available at [33].

### 2.2. Satellite Budget Uplink

**Figure 7:**
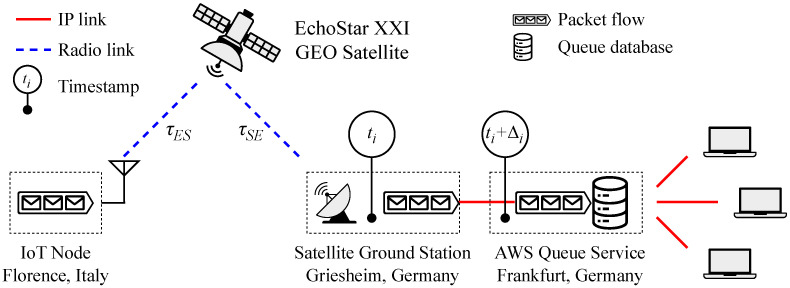
System block diagram. The IoT node—located in Italy during the experiments—transmits a package to the EchoStar XXI GEO satellite. This relays the package to the satellite gateway in Griesheim, Germany. Up to this point, the communication is on a radio link and from this point on it is on an IP link. In the satellite gateway, the package is decoded, timestamped and forwarded to AWS. When the decoded message arrives in AWS, it is timestamped and made available for access.

The block diagram of the IoT system is provided in Figure 7.

Connectivity across Europe is provided by the GEO satellite EchoStar XXI (https://echostarmobile.com/about/coverage/ (accessed on 4 August 2025)), which relays the packages received from the IoT node to the satellite gateway ground station in Griesheim, Germany. From there, the transmitted messages are forwarded to the cloud service, located in Frankfurt, Germany. Among the elements shown in Figure 7, only the IoT node and the cloud service interface were developed by the authors; the remaining components are managed by EchoStar Mobile Ltd or Amazon Web Services (AWS).

The packets transmitted by the IoT node undergo a propagation delay of $\tau_{ES}\approx 120\;\mathrm{ms}$, they are relayed by the GEO satellite, and reach the satellite ground station after another propagation delay $\tau_{SE}\approx \tau_{ES}$. The packet is then demodulated by the gateway, timestamped at instant $t_{i}$, and forwarded to the cloud service. There it is again timestamped at instant $t_{i}+\Delta_{i}$ and then stored in the queue of the cloud service.

The radio link between satellite and ground station is managed by EchoStar Mobile Ltd. The link budget between the IoT node and the satellite is given by [36]

(1) $$P_{r}=\mathrm{EIRP}+G_{r}-L_{fs}-L_{p}-L_{a}-L_{o}$$

where $P_{r}$ is the received power at the satellite end, the EIRP of the IoT node is the sum of the transmitted power and the transmitting antenna gain, $G_{r}$ is the satellite antenna gain, $L_{fs}$ is the free space path loss, $L_{p}$ is the loss due to polarization misalignment, $L_{a}$ is the atmospheric loss, and $L_{o}$ accounts for additional losses.

In Equation (1), the EIRP is adaptively changed to reduce power consumption while assuring a successful communication, and it is then considered as an operative parameter. The free space loss is evaluated as $L_{fs}\left[dB\right]=20\log_{10}\left(4\pi d/\lambda \right)$, with *d* the satellite to IoT node distance (approximately 3.6 × 10^4^ km) and λ≈0.15 m the wavelength at the working frequency: substituting these values, we have $L_{fs}=190\;\mathrm{dB}$. The GEO satellite antenna is a deployable dish antenna with a diameter of 18 m (https://www.disk91.com/2025/technology/lora/echostar-iot-the-geostationary-lorawan-solution-for-europe/ (accessed on 4 August 2025)). Using the simplified formula for parabolic antennas, the gain is $G_{r}\left[dB\right]=20\log_{10}\left(D/\lambda \right)\approx 49\;\mathrm{dBi}$ with *D* the diameter of the antenna. The parameter $L_{o}$, representing loss due to other causes, is difficult to estimate. Polarization loss is $L_{p}=3\;\mathrm{dB}$ because the IoT node antenna is linearly polarized, and the GEO satellite antenna is circularly polarized. Atmospheric loss and additional losses are estimated to be $L_{a}=0.5\;\mathrm{dB}$ and $L_{o}=1\;\mathrm{dB}$ [36].

Eventually, substituting these numerical values into Equation (1), the estimated link budget becomes $P_{r}=\mathrm{EIRP}-144\;\mathrm{dB}$. For a successful communication, the link budget must be greater than the sensitivity S=−134.5 dBm (the greater value for DR9 from Table 2 is taken as worst case scenario). Then,

(2) $$P_{r}=\mathrm{EIRP}-144\;\mathrm{dB}>S=-134.5\;\mathrm{dBm}\Rightarrow \mathrm{EIRP}>9.5\;\mathrm{dBm}$$

The EIRP condition reported in Equation (2) is consistent with the RMU capability of providing a maximum EIRP of 27.5 dBm with an antenna input power of 27 dBm. There is then a margin for the RMU antenna input power, which can then be used to adapt to variable operating conditions, e.g., misalignment between an IoT node and GEO satellite antennas, bad weather, and the presence of obstacles such as trees in the line of sight.

### 2.3. Cloud Service and Python Application Programming Interface

The cloud service was implemented using AWS (https://aws.amazon.com/ (accessed on 4 August 2025)). In this framework, messages transmitted by the IoT node are stored in queues, which must be periodically polled for access and visualization. Within the LoRa network, each queue is uniquely associated with the serial number of the transmitting RMU module. As a result, the data retrieved from the cloud can be reliably mapped to the corresponding IoT node, thereby enabling clear differentiation among multiple nodes. During the preliminary tests, two IoT nodes were operated simultaneously, and their distinction was ensured through the serial numbers of the RMUs. In the experiments reported below, however, only a single IoT node was active at any given time.

The messages stored in AWS are encoded in base-64 format and must be converted to ASCII for interpretation. Each message is also assigned a unique identifier by AWS. Message processing in AWS is carried out through a Python script (Python version 3.11.5) that employs the *boto3* library (https://boto3.amazonaws.com/v1/documentation/api/latest/index.html version 1.40.53 (accessed on 4 August 2025)) to access AWS services and execute the following steps

1. start a communication with AWS;
2. read messages in the queue;
3. delete messages in the queue;
4. decode the messages;
5. get data;
6. process the data in the messages.

Supplementary Material for this script is available at [33].

In Table 4, the collected sensor parameters are reported, including ASCII character field lengths and an example from experimental tests. Some parameters require an offset to be added to the raw sensor reading, before transmission, to prevent negative values, as outlined in Table 4. According to the datasheet of the RMU, the maximum payload size is 51 bytes. Transmitting in base-10 requires converting the data into ASCII characters, resulting in 58 characters, exceeding 51. Decimal points are thus removed, and the characters are combined into a single base-10 string, which is then converted into 34 base-64 digits (26 bytes), thus under the 51-byte limit. The data from the current probe are 12 base-64 digits (9 bytes), for a total of 46 base-64 digits (35 bytes). This is passed directly to the RMU using the AT + SENDB command. The base-16 version of Table 4 example is

04f8f46da071ef7bb6072e1a28d4072c1c820a4040116015b0

which converts to base-64 in the satellite communication stack as

BPj0baBx73u2By4aKNQHLByCCkBAEWAVsA==

The physical bit rate for LR-HFSS modulation is either 162 bit/s or 325 bit/s for DR 8 and DR9, respectively, (Table 2). The time on air for the 35 byte-payload is thus approximately 2.6 s for DR8 and 1.4 s for DR9 [35]. The time on air is a key feature as in the LR-FHSS modulation it dominates the latency from the packet transmission to the message queuing in the cloud service [37] (Figure 7).

## 3. Results

The experimental results of the IoT node are given in terms of EIRP, data from the sensors, data of the communication between the RMU and the GEO satellite, and drained battery charge. Except for EIRP, two tests were carried out: (i) *standing test* in which the IoT node remained still for a long duration (258 min); (ii) *moving test* in which the IoT node was carried in the hand of a pedestrian for a short duration (63 min). The tests was intended to verify the proper working of the IoT node and to assess how the IoT node performs in terms of power consumption and delay between gateway and cloud service on real-world experimental tests. Although the test areas were covered by other communication services, these did not affect the operation of the system. Therefore, testing satellite connectivity for remote areas can be effectively carried out, even in urban environments [17].

### 3.1. Characterization of the Antenna Transmission

The simulated antennas within the enclosure are shown in Figure 6. The dielectric permittivity and loss tangent of ABS were measured with a cavity method [38] at 4.24 GHz, yielding values of 2.25 and 0.06, respectively. The radiation performance of the RMU transmitter was first investigated through simulations using the frequency-domain solver in ANSYS Electronics Desktop (https://www.ansys.com/ version 2024 R2 (accessed on 4 August 2025)). The antenna model, provided by the manufacturer, that is, Kyocera Corporation, headquartered in Kyoto, Japan, was integrated onto a PCB for both the Tx and Rx antennas, as illustrated in Figure 6. The antennas were fed through coplanar waveguides with ground vias. Antenna tuning was achieved using LC networks, with component values estimated from simulations: 5.6 pF and 1.6 nH for the Tx antenna, and 5.6 pF and 0.1 nH for the Rx antenna.

**Figure 8:**
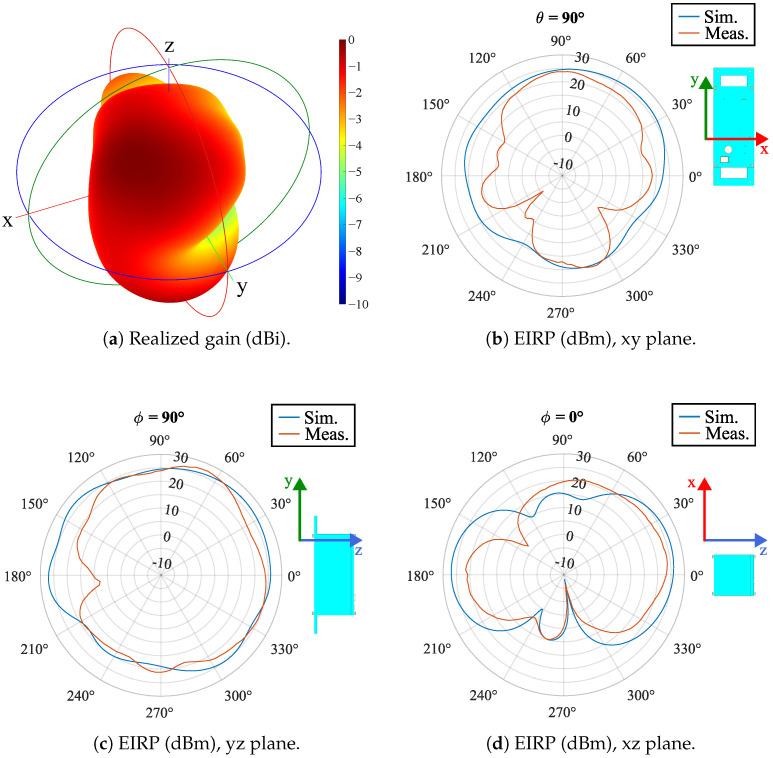
Tx antenna characterization at 2.00 GHz: (**a**) simulated normalized realized gain in dBi (the maximum gain is 0.45 dBi for θ=42° and ϕ=44°); (**b**) EIRP in dBm on θ=90° for the polarization along ϕ (E-plane); (**c**) EIRP in dBm on ϕ=90° for the polarization along ϕ (H-plane); (**d**) EIRP in dBm on ϕ=0° for the polarization along θ. In (**b**–**d**), the CAD model of the IoT node is depicted to represent its orientation. For the EIRP, the input power is 27 dBm.

The Tx antenna was measured in an anechoic chamber. The N1996A spectrum analyzer from Agilent Technologies Inc., Santa Clara, CA, USA, was used. The probe antenna is the HyperLOG^®^ 7060 from Aaronia AG, Strickscheid, Germany. During EIRP measurements, the RMU was controlled by a dedicated MCU to drive the antenna in continuous wave with an input power of 27 dBm. In simulations, the same input power is added to the realized gain to get the EIRP. The simulated 3D radiation pattern is reported in Figure 8, whereas the 2D simulated and measured EIRP for the Tx antenna inside the box are shown in Figure 8b–d.

### 3.2. Sensor Data

**Figure 9:**
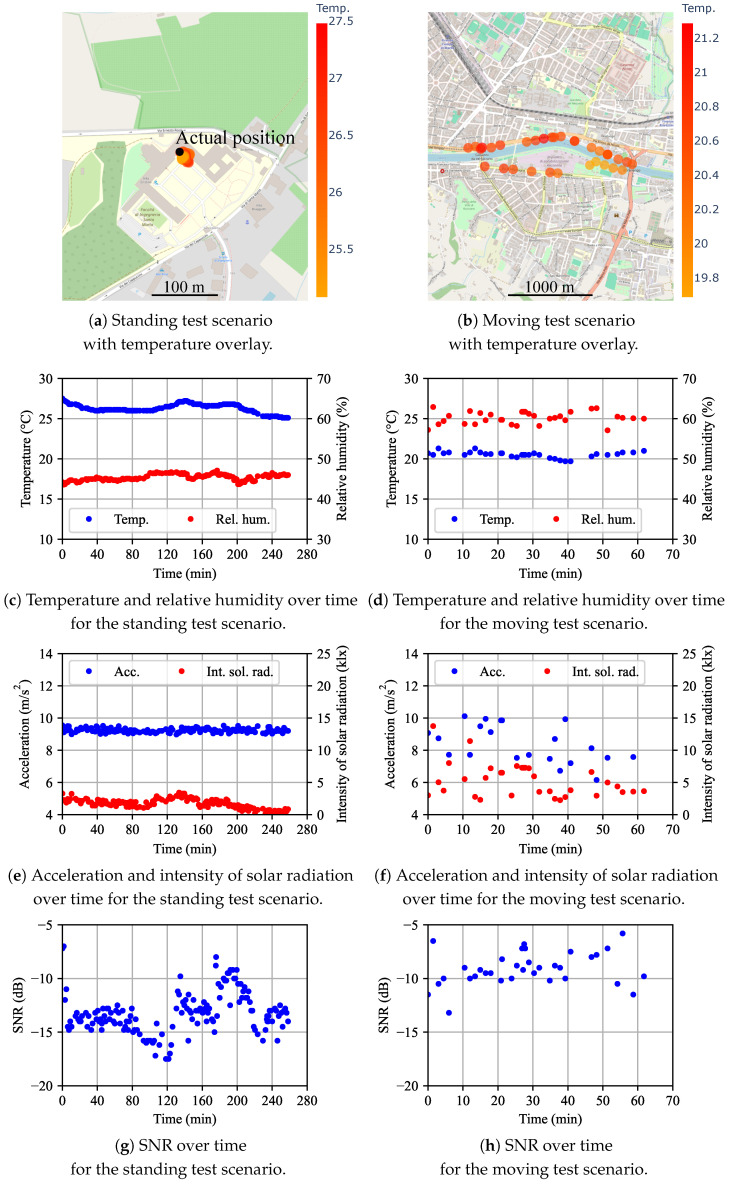
Standing (**left**) and moving (**right**) tests. Start time (time = 0 min) in the GMT time zone for the standing (moving) test is Monday 26 May 2025 13:10:02.823 (GMT+02:00 DST) [Monday 26 May 2025 08:41:44.589 (GMT+02:00 DST)].

The data collected from the two tests are summarized in Figure 9, including temperature, humidity, acceleration magnitude, solar radiation intensity, and signal-to-noise ratio (SNR). In the standing test, the IoT node was placed on the laboratory windowsill at Via di Santa Marta 3, I-50139 Firenze, Italy [black dot in Figure 9a]. The uplink radio link quality was evaluated through the SNR, as reported in Figure 9g,h.

### 3.3. Current Absorption

**Figure 10:**
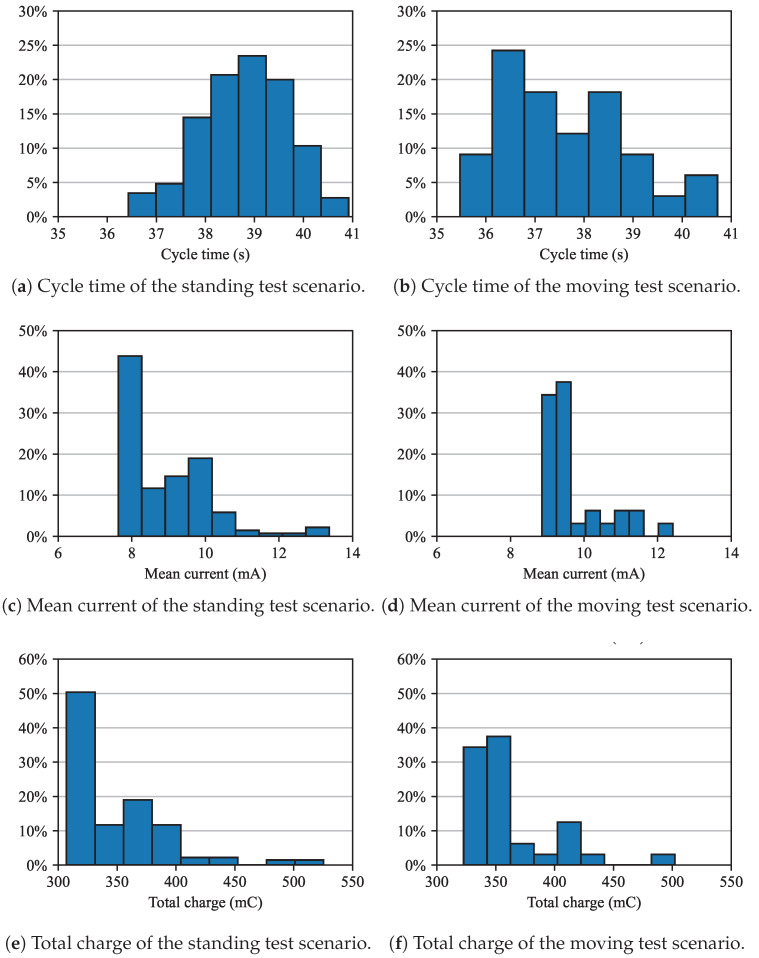
Measurements from the current probe in the form of histograms with outliers removed: standing test (**left**) and moving test (**right**).

The supply current was sampled at 100 Hz, a rate too high for real-time transmission via satellite. Instead, the cumulative sum of the current samples, together with the corresponding number of time counts for each cycle, was transmitted. From these data, the total charge drained per cycle and the cycle duration were derived. The results for cycle duration, mean current, and total charge are presented in Figure 10 for both standing and moving tests. Data with outliers removed are also shown in aggregate form as histograms. Outliers were identified as follows: the standard score (z-score) was computed as z=(x−μ)/σ, where *x* is the original value, μ the mean, and σ the standard deviation. Values with a z-score greater than one were considered outliers and excluded from the analysis.

To eliminate the systematic offset error introduced by the current probe, a calibration procedure was applied. Specifically, the mean current was first measured during the transmitting state (40 s), after which the RMU was switched to the sleep state for the same duration. The mean current in the sleep state was then subtracted from that of the transmitting state. The results reported in Figure 10 refer only to the transmitting cycle, although the complete calibration sequence spans 80 s.

**Figure 11:**
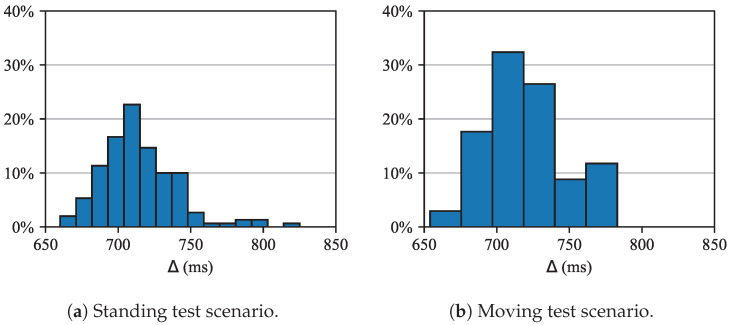
Delay between gateway and cloud service, Δ [Figure 7]: standing (**a**) and moving (**b**) test.

Figure 11 reports the communication delay, Δ, between the satellite gateway and the cloud service for both test scenarios. The histogram distributions are similar for both scenarios, except for a longer right tail in the standing test, likely attributable to weather conditions during the experiment. The average delay across both scenarios is 717 ms.

## 4. Discussion

### 4.1. Characterization of the Antenna Transmission

In terms of transmission capability, the maximum simulated gain in Figure 8a has a value of only 0.45 dBi for θ=42° and ϕ=44°, whereas a maximum measured EIRP of 27.5 dBm was observed on the H-plane in Figure 8c for θ=66°. As the input antenna power is 27 dBm, the maximum measured realized gain is 0.5 dBi, in line with the simulations. A low value for the realized gain implies an almost omnidirectional pattern, a feature expected for an IoT node as the orientation of the IoT node is not controlled in real applications and it is then unaware of the position of the receiving antenna.

The radiation pattern [Figure 8] does not guarantee good coverage for each orientation between the IoT node and the GEO satellite, as the measured data reveal few blind directions, at θ=90°, ϕ≈215° and θ≈280°, ϕ=0°, the latter captured also by simulations.

While the subsystems layout in the IoT node is still preliminary, we have verified that the proposed configuration does not impact on the radiation performance of the RMU.

### 4.2. Sensor Data

Let us now discuss the *standing test*. In this test, the node was positioned in a fixed location [Figure 9a]; however, there is a discrepancy in the localization. The achieved accuracy and precision, on the order of one meter, are considered adequate for most traceability applications in agrifood supply chains. The number of satellites in line of sight to the GNSS module varied between 5 and 13.

The temperature and humidity for the standing test are visible in Figure 9c. They are stable over time. The temperature ranges from 25.1 °C to 27.5 °C. It should be noted that no special attention was given to sensor placement, such as shielding from direct sunlight. This explains the apparent inaccuracy in the temperature measurements. The humidity is in the 43.6–47.1% range, a variability that can be attributed to environmental conditions rather than sensor accuracy.

The acceleration for the standing test is shown in Figure 9e. The mean value and standard deviation are 9.24 m/s^2^ and 0.13 m/s^2^, respectively. The mean value is different from the expected value of 9.81 m/s^2^, mainly due to the systematic offset error. The latter is due to contributions that slightly modify the factory calibration, such as thermal stress during IC soldering and misalignment of the accelerometer package relative to the coordinate system. Improved results may be achieved with a more accurate calibration.

The intensity of solar radiation for the standing test, reported in Figure 9e, was almost constant during the test; the day of the test was cloudy with diffused daylight. As the sensor pattern is almost conic, even at midday, the intensity of sensed solar radiation may be low if the sensor does not point directly to the Sun.

The SNR of the uplink path, from the IoT node to the satellite, is reported in Figure 9g for the standing case. Its SNR ranges from −17.5 dB to −7.0 dB, which is significantly higher than the minimum required, that is, −20 dB, to establish the link.

Let us now focus on the *moving test*. The path for this test is drawn in Figure 9b. Observe that the IoT node was moved along the bank sides of a river, in an open area. The sampling density is not uniform along the path because some messages were lost. The number of satellites visible to the GNSS module ranges from 9 to 20. These values are higher than those for the standing test, likely because the test environment presented fewer obstacles in the line of sight with the GEO satellite.

In the moving test, the temperature variation in Figure 9d is less than in the standing test and goes from 19.7 °C to 21.3 °C. Similar considerations can be made for humidity, ranging from 57.1% to 62.9%. On the other hand, the acceleration for the moving test in Figure 9f oscillates due to the pedestrian gait oscillations.

Concerning the intensity of solar radiation in Figure 9f, it was slightly cloudy during the test. Indeed, the values for the intensity of solar radiation are greater than for the standing test. However, the values oscillate more because of the shadows generated by vegetation and the pedestrian carrying the IoT node.

The SNR for the moving test, shown in Figure 9h, ranges from −13.2 dB to −5.8 dB, exceeding the values observed in the standing test, likely due to improved line-of-sight visibility to the satellites.

### 4.3. Current Absorption

During tests, a nominal cycle time of 40 s was chosen, which is composed of sensor readings, message sending, and a sleep period. However, the distributions of the cycle time, reported in histograms for both standing and moving tests in Figure 10a,b, show that the actual cycle time shows some dispersion, and its mean is lower than the set value. This is most likely due to a non-calibrated MCU clock reference.

The mean RMU supply current, shown in Figure 10c,d, was calculated by integrating the instantaneous current—excluding the sleep-state contribution—and dividing by the cycle duration; this procedure was repeated for each packet. The results indicate a slight discrepancy between the two tests, with mean values of approximately 8 mA and 9.2 mA, respectively.

The total drained battery charge per cycle is reported in Figure 10e,f. The mean for the total charge drained over both tests is approximately 356 mC. On this basis, an estimate of the number of cycles that can be handled by the specific battery pack provided is given below

(3) $$N_{c}=\frac{Q_{B}}{Q_{c}}=\frac{2500\;\mathrm{mAh}}{356\;\mathrm{mC}}=\frac{2500\;\cdot \;3600\;\mathrm{mC}}{356\;\mathrm{mC}}\approx 25.3\times 10^{3}$$

where $N_{c}$ is the number of cycles, $Q_{B}$ the battery charge, $Q_{c}$ the charge per cycle, and 1 mAh=3600 mC. The battery life depends on the message frequency as follows:

(4) $$L_{B}=\frac{N_{c}}{f_{M}}$$

where $L_{B}$ is the battery life and $f_{M}$ the message frequency. The data rate requested in the typical use case is low. In [25], a 5 min interval for cattle tracking is considered. This interval corresponds to a message frequency of $f_{M}=0.0033\mathrm{Hz}$ and a battery life of approximately 88 days. Besides periodic data transmission, other approaches were adopted for efficient energy management, such as event-triggered transmission [39].

Based on the mean total charge drained, 356 mC, a supply voltage of 3.3 V, and a payload size of 280 bits, the energy cost per transmitted bit is calculated as E=356 mC·3.3 V/280 bit=4 mJ/bit, which is consistent with the values reported in the literature [32]. Considering the packet as a whole, the energy per packet is $E_{p}=356\;\mathrm{mC}\;\cdot \;3.3\;V=1.2\;J$. When the packet error rate, $e_{r}$, is taken into account, the energy per successfully transmitted packet is given by $E_{ps}=E_{p}/\left(1-e_{r}\right)$. Consequently, the energy efficiency—defined as the ratio between the energy per transmitted packet and the energy per successfully transmitted packet—is $\eta =E_{p}/E_{ps}=1-e_{r}$. Thus, higher packet error rates lead to lower energy efficiency in packet transmission.

The above estimate assumes that the current of the RMU in sleep mode was negligible; actually, its value is 30 μA (Table 1). It turns out that this additional drained charge is more relevant in the case of low data rate demands. Not least, the RMU is the most power-hungry component of the realized IoT node prototype; nevertheless, all other sensors and modules, first of all the MCU, absorb a quantity of current that could be non-negligible in a very low data rate profile of use. Consequently, the effective battery life estimated in (3) should be considered as an upper limit.

**Figure 12:**
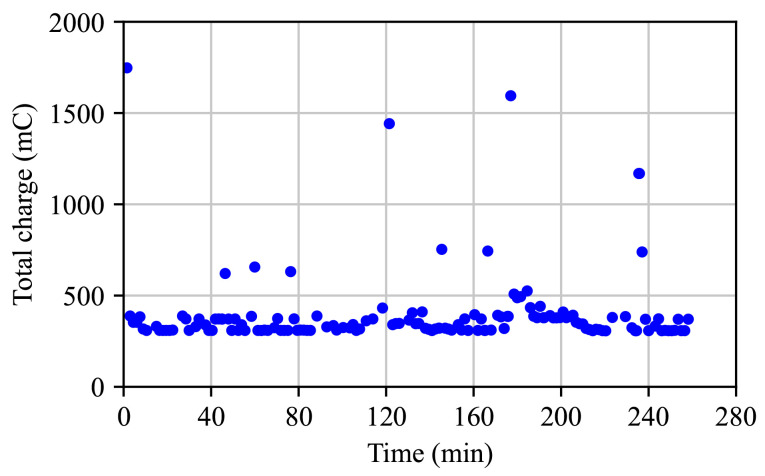
RMU drained charge per packet-sending cycle, during the standing test.

Figure 12 reports the battery charge drained per packet-sending cycle. The mean value, 356 mC, corresponds to a standard single-packet transmission under the considered use-case conditions. Several outliers are also observed. Those in the ranges 650–750 mC and 1000–1100 mC indicate that two and three transmission attempts, respectively, were required due to lost ACK responses in previous attempts. The remaining three outliers, at even higher charge values, correspond to cases in which the MCU repeated the RMU joining procedure after reiterated failures.

The main results discussed in this section are summarized in Table 5, providing a concise overview of the key performance metrics for the IoT node under both standing and moving test conditions. The table highlights parameters such as test duration, cycle duration, gateway-cloud service delay, and total charge per cycle, allowing for a direct comparison between the different operational scenarios. In addition, the mean values over both test scenarios are reported. This summary facilitates a clear understanding of the IoT node, and supports the subsequent discussion on practical deployments.

### 4.4. Network Scalability

The FHSS technique aims to reducing collisions between packets, thus enhancing the network scalability for both terrestrial and satellite networks [19]. To provide a quantitative insight on the network reliability and scalability, according to system performance from Semtech, Ref. [37] the network load amounts to approximately 1 million of packets per day with an admissible packet error rate of 10%. This value is intended per gateway and per satellite beam, as provided by the EchoStar XXI satellite coverage (https://echostarmobile.com/about/coverage/ (accessed on 4 August 2025)) (the number of beams for the EchoStar XXI satellite is 182).

To provide a numerical estimate of the RMUs that can coexist, provided that a 5 min interval for cattle tracking is considered as in [25], the packets transmitted per RMU per day are 288. The number of RMUs that can then transmit in a day are approximately $10^{6}/288\approx 3500$. Again, this value is intended per gateway and beam.

The above estimation indicates the number of RMUs that can be managed during one day, but it provides no clue about the number of RMUs that can transmit simultaneously. For an insight on this, we must rely on the network capacity, defined as *the total number of data bits received correctly by the gateway per second when the packet receiving ratio is 0.9* [19]. According to [19], for a LEO non-terrestrial network, the network capacity is 4.04 kbps and 3.29 kbps for DR8 and DR9, respectively. Similar values are expected for a GEO non-terrestrial network, as packet loss is mainly caused by collision if the SNR is high enough [19]. Eventually, the number of RMUs that can transmit simultaneously is obtained by dividing the network capacity provided above by the physical bit rate in Table 2, obtaining 25 and 10 for DR8 and DR9, respectively.

The figures provided above for the maximum number of RMUs manageable in a day and the maximum number of RMUs that can transmit simultaneously can be increased by increasing the number of gateways and by improving the digital signal processing [37]. For more details related to collision and scalability and for techniques aimed at improving the network capacity, the reader should refer to [18,40,41,42].

### 4.5. Cost Analysis

The cost-effectiveness of the proposed system represents a critical consideration, as elevated monitoring expenses would inevitably influence the final price of agrifood products for end-consumers; while a detailed cost analysis is beyond the scope of this work, the main cost items associated with GEO satellite communications can be identified and contrasted with those of terrestrial long range wide area network (LoRaWAN) and LEO satellite systems. Three principal cost items are involved: (a) hardware, primarily the RMU; (b) software, including subscription fees for cloud-based services; and(c) connectivity, represented by subscription fees for GEO satellite services, in cases where licensed spectrum is employed. The first two cost items are identical for terrestrial LoRaWAN and LEO satellite solutions, whereas the third may be different for GEO satellite communications.

## 5. Conclusions

In this paper, we presented an IoT node employing LoRa GEO satellite connectivity, specifically designed for PA applications. Satellite connectivity is enabled through a dedicated commercial RMU operating in the S-band with LR-FHSS modulation. The IoT node was experimentally tested in real-life scenarios, demonstrating its effectiveness. To illustrate the potential of such a system, the prototype was equipped with a set of conventional low-power sensors suitable for cattle tracking. Sensor data were collected and transferred to the RMU by a low-power MCU, thereby demonstrating the feasibility of LoRa GEO satellite connectivity in IoT-oriented applications, particularly in PA scenarios. It should be noted, however, that no actual tests on livestock were performed.

In terms of energy requirements, the RMU proved to be the most power-demanding subsystem of the DtS IoT prototype. Its charge consumption per transmitted packet was measured during the experimental campaign, yielding an estimate of 356 mC. With the adopted battery pack (four 2500 mAh NiMH cells), this corresponds to an upper bound of about 25 × 10^3^ transmission cycles. This estimate, however, accounts only for the RMU and does not include the remaining components of the IoT node.

The outcomes of this work highlight that GEO DtS IoT node development requires significant effort in system integration, ad hoc sensor set design, and careful selection of the MCU. At present, the communication technology itself does not represent the critical part of the radio subsystem. Nevertheless, the design of transmitting and receiving antennas—possibly integrated into the IoT node enclosure with beam-pointing diversity—would improve the reliability of the communication link.

In conclusion, we assess that LoRa technology can be adopted to integrate terrestrial and non-terrestrial IoT communications. The availability of multi-band LoRa transceivers, combined with suitable antenna systems, will enable future developments that extend existing IoT networks with remote-area coverage capabilities.

## Figures and Tables

**Table 1:** Current consumption for the RMU in every operative state, data from [10].

State	Symbol	Measured Current (mA)
Sleep	$i_{\mathrm{sl}}$	0.030
Idle	$i_{\mathrm{id}}$	10
Receiving (ACK)	$i_{A}$	70
Transmitting ($P_{TX}=15\;\mathrm{dBm}$ ^(1)^)	$i_{S}$	100 ^(1)^
Joining ($P_{TX}=27\;\mathrm{dBm}$)	$i_{S}$	230

^(1)^ The value of $P_{TX}$ changes adaptively to assure a successful communication with the minimum amount of power. The respective current consumption changes accordingly.

**Table 2:** Parameters for the LR-FHSS modulation. The values for occupied bandwidth per hop and physical bit rate are from [18], while the sensitivity is from [34] and the time on air from [35].

Parameter	Value		
Frequency	2.009 GHz + {453.0, 609.4, 765.6, 921.9} kHz		
Operating channel width	137 kHz		
Occupied bandwidth per hop	488 Hz		
Data rate	DR8		DR9
Code rate	1/3		2/3
Physical bit rate (bit/s)	162		325
Sensitivity (dBm)	−137		−134.5
Time on air [35 byte payload size] (s)	2.6		1.4

**Table 3:** Maximum supply current of every hardware component in the IoT node.

Module	Max. Curr. (mA)
GNSS module	60
Accelerometer	0.15
Temperature and humidity sensor	1.50
Intensity of solar radiation sensor	0.50
RMU (joining state)	230
MCU	500

**Table 4:** Data transmitted in each package with maximum ASCII characters required, offsets, and results in base-10 for the example. The quantities are zero-padded for the required field length.

Sensor	Datum	ASCII Chars	Offset	Ex. Base-10
GNSS	Latitude	10	0900000000	0434789850
	Longitude	10	1800000000	0111520182
	Number of satellites	2	00	07
	Altitude	5	10000	01802
	Height	5	10000	00452
Accel.	Acc. x	5	16384	00064
	Acc. y	5	16384	−11936
	Acc. z	5	16384	−10832
Temp. and hum.	Temperature	3	300	156
	Humidity	3	000	522
Solar rad.	Int. solar radiation	5	00000	01836

**Table 5:** Mean values for the results of the two tests. The repetition for the packet transmission is 80 s.

Parameter	Standing Test	Moving Test	Overall
Duration (min)	258	63	–
Cycle time (s)	38.8	37.6	38.2
Gateway-cloud service delay (ms)	714	719	717
Total charge per cycle (mC)	347	364	356

## Data Availability

The original data presented in the study are openly available in GitHub at https://github.com/Gianne97/IoTnodeWithGEOconnectivity/ DOI: 10.5281/zenodo.16738961.

## Abbreviations

The following abbreviations are used in this manuscript:

ABS	acrylonitrile butadiene styrene
ACK	acknowledgement
AWS	Amazon Web Services
DtS	direct-to-satellite
EIRP	effective isotropic radiated power
GEO	geostationary Earth orbit
GNSS	global navigation satellite system
IC	integrated circuit
IoT	Internet of Things
ISM	industrial, scientific and medical
LEO	low Earth orbit
LR-FHSS	long-range frequency hopping spread spectrum
LoRa	Long Range
LoRaWAN	long range wide area network
MCU	microcontroller unit
NGEU	Next Generation EU
NRRP	National Recovery and Resilience Plan
PA	precision agriculture
PCB	printed circuit board
RMU	radio-modem unit
SNR	signal-to-noise ratio
